# Mainstreaming production and nutrient resilience of vegetable crops in megacities: pre-breeding for terrace cultivation

**DOI:** 10.3389/fpls.2023.1237099

**Published:** 2023-11-17

**Authors:** Kun Ma, Yuan Yuan, Caochuang Fang

**Affiliations:** Shanghai Key Laboratory of Protected Horticultural Technology, Horticultural Research Institute, Shanghai Academy of Agricultural Sciences, Shanghai, China

**Keywords:** pre-breeding, genetic improvement, allele, terrace vegetables, resilience

## Abstract

Modern megacities offer convenient lifestyles to their citizens. However, agriculture is becoming increasingly vulnerable, especially during unexpected public health emergencies such as pandemics. Fortunately, the adaptability of terrace vegetables cultivation presents an opportunity to grow horticultural crops in residential spaces, bringing numerous benefits to citizens, including enhanced nutrition and recreational engagement in the cultivation process. Although certain planting skills and equipment have been developed, the citizens tend to sow some seeds with unknown pedigree, it is rare to find new plant varieties specifically bred for cultivation as terrace vegetables. To expand the genetic basis of new breeding materials, elite parents, and varieties (pre-breeding) for terrace cultivation, this review not only discusses the molecular breeding strategy for the identification, creation, and application of rational alleles for improving horticultural characteristics including plant architecture, flavor quality, and ornamental character, but also assesses the potential for terrace cultivation of some representative vegetable crops. We conclude that the process of pre-breeding specifically for terrace cultivation environments is vital for generating a genetic basis for urban terrace vegetable crops.

## 1 Introduction

Terrace cultivation had existed since the days of Babylon in 2300 BC (Udayan and Sreedaya, 2018). While in modern societies, terraces/balconies are the only places that citizens could cultivate their own vegetables with the expanding of megacities (Chitra, 2021), so local nutrient sources are rapidly depleting, consequently hindering food supplies. Moreover, as the COVID-19 pandemic subsides, challenges to food supplies induced by public health disturbances suggest that urban nutrient resilience may be crucial for meeting the needs of megacities (Langemeyer et al., 2021). Urban nutrient resilience reflects the sustainability of cities, which is based on resource availability and the need to enhance food supplies and quality of life (Zeng et al., 2022). Fortunately, terrace cultivation of vegetable crops plays a crucial role in urban nutrient resilience as it not only strives to provide food daily or emergently, but also enhances recreation in high-density cities. Additionally, in many countries such as USA, India, China, and Korea, terrace cultivation is becoming more and more popular among the citizens, and many cultivations equipment or materials are sales good.

The terrace cultivation of vegetable crops is characterized by utilizing edge/corner vacancies or installing box/shelf-shaped equipment on terraces, balconies or roofs of high-density residential buildings for the purpose of planting and growing vegetables. For this, complete commercialized cultivation substrates have been matched adequately for cultivation of vegetables (Kader et al., 2022). Many planting modes can be utilized on balconies or roofs, including placing pots along terrace handrails, utilizing compact versions of vertical farming equipment, employing light-emitting diode (LED) photon boxes, utilizing hollowed walls, creating extended grooves, and using slope shelves with aerosol cans. Thus, it is a half-open, half-protected, half-controlled, flexible, and relatively cramped cultivating environment. To adapt to this environment, three prerequisites are required for the cultivated plants and surely for the germplasm: First, adaptation to the limited planting space, which is a major limiting factor. The plant architecture should be reduced to a size that can be accommodated by terraces, balconies or roofs. Second, to enhance nutrient supply to citizens, it is necessary to ensure high levels of nutrient content and flavor quality. Terrace vegetables have the potential to independently produce rare, fresh, more nutritional, more flavorful, and higher value-added vegetable products. This eliminates the need for logistics processes and helps address urgent disruptions in nutrient supply caused by delays in vegetable transportation due to a pandemic or other social reasons, i.e., terrace vegetable cultivation contributes to increasing urban nutrient resilience. Third, being connected to the human environment, the ornamental value of vegetable germplasms should also be considered to complement the existing urban landscape ecology and satisfy the preferences of citizens engaged in terrace vegetable cultivation.

To obtain germplasms with the specialized attributes required for terrace vegetable systems, we propose the use of pre-breeding strategy as a bridge to connect genetic variations with breeding programs (Akkenapally and Kumar, 2022). Pre-breeding refers to all activities designed to identify desirable characteristics and/or genes from unadopted (exotic or semi-exotic) materials, including those that, although adapted have been subjected to any kind of selection for improvement (Akkenapally and Kumar, 2022). This strategy allows for the combination of valuable artificial or natural alleles into the recipient material, resulting in the development of highly beneficial germplasms. The aim of this review is to summarize an integrated pre-breeding strategy for expanding terrace vegetable germplasms by means of showing some examples of vegetable crops.

## 2 General breeding traits and breeding methods for terrace vegetables

Compact plant architecture is the highest priority breeding target for terrace vegetables. Under this premise, lots of vegetable crops have the potential for use in terrace vegetable systems, while certain plants do not possess traits that align with the three prerequisites. For instance, the size of most fruit vegetable crops exceeds the available space on a terrace. Meanwhile, although some germplasms exhibit a compact plant architecture, they may not yield as highly or adapt well to continuous harvest which might be eliminated. While for most leafy vegetables and root vegetables, their plant heights naturally suitable for terrace environments, so the flavor quality, nutrient quality, and ornamental value should be more concerned in pre-breeding programs. The evaluation of adaptability and deficiency of different vegetables for terrace cultivation were listed in **Supplementary Table 1** as examples for reference.

The 5G breeding is an approach that fully reflects the newest modern molecular breeding spirits; these 5Gs are 1^st^ G Genome assembly, 2^nd^ G Germplasm characterization, 3^rd^ G Gene function identification, 4^th^ G Genomic breeding (GB), and 5^th^ G Gene editing (GE) (Varshney et al., 2020). Combining the concepts of pre-breeding, the clustered regularly interspaced short palindromic repeats (CRISPR) is included by the 5^th^ G, the quantitative trait locus (QTL) mapping is included by the 2^nd^, 3^rd^ and 4^th^ G, the genome-wide associated study (GWAS) is included by the 2^nd^ G, and the marker assisted selection (MAS) is included by the 4^th^ G, which are all commonly used molecular breeding technologies that can be employed to improve specific characteristics of crops, according to the purpose of pre-breeding.

## 3 Tomatoes genetic improvement exhibits a representative of pre-breeding strategy for terrace cultivation

Tomato is one of the most popular fruit vegetable crops around the world in a long history (Razifard et al., 2020). However, the plant architecture of most tomato cultivars is relatively taller for terrace cultivation. In tomatoes, three main effective genes are useful for molding compactness in the plant architecture (Kwon et al., 2019): *SISP*, *SISP5G*, and *SLER*. The gene *SlSP* has been found to delay flowering time and promote indeterminate growth, *SlSP5G* delays flowering time especially under long-day conditions, and *SLER* promotes the elongation of internodes (Torii et al., 1996; Pnueli et al., 1998; Xu et al., 2015; Soyk et al., 2016). Mutations in *SlSP*, whether naturally occurring or induced by CRISPR, result in a determinate growth habit without yield loss, while mutating of *SlSP5G* accelerates flowering in long-day conditions (Pnueli et al., 1998; Soyk et al., 2016). Additionally, the natural *Sler*, mutant shows shortened internodes and extremely compact inflorescences, forming tight fruit clusters (Kwon et al., 2019). The double-mutated genotype *Slsp/Slsp5g* exhibits rapid cycling and compacts the plants without affecting fruit number or yield when cultivated in high-density planting spaces (Soyk et al., 2016). The triple mutant *Slsp/Sp5g/Sler* is the most compact and exhibits significantly decreased fruit weight and yield than the *sp* mutant in the Mo82 background. Fortunately, the triple mutant has similar fruit numbers per plant, Brix content, and yield when planted in high-density LED-assisted photon greenhouse fields compared with those exhibited by the *sp* mutant in the Sweet100 background, indicating that the triple mutated genotype is most suitable for tomato terrace planting (Kwon et al., 2019).

Flavor quality, being the foundation of edibility, represents crucial targets for pre-breeding efforts (Bomgardner, 2017). The utilization of CRISPR mutants of *SlINVINH1* and *SlVPE5* resulted in increased glucose, fructose, and Brix values, raising levels of 40.82%, 42.76%, and 32.76, as well as 35.83%, 43.0%, and 32.43% than the parental lines, respectively (Wang B et al., 2021). These improvements were achieved without any significant alteration in fruit weight (Wang B et al., 2021; Kawaguchi et al., 2021). A genome-wide association study (GWAS) identified a main effect quantitative trait locus (QTL) located on chromosome 9 at position 62.64 M, which was found to explain 28.73% of the phenotypical variation observed during a 3-year test (Kim et al., 2021). Additionally, conditional QTL for taste quality should be explored and used as modern terrace -cropping often uses photon suppliers such as HPS and LED lamps. In a QTL mapping analysis of Brix values, two QTL regions have been identified; the first QTL was located on chromosome 2 at 43.5 - 50.5 Mb under both HPS and LED conditions, explaining 20% of the phenotypic variance; and the second QTL was found on chromosome 6 at 43.7 - 47.1 Mb only under the LED condition, explaining 26% of the phenotypic variance (Prinzenberg et al., 2021). The additive effect of these QTLs suggests that stacking high flavor quality alleles could lead to continuous improvement in Brix values in elite breeding lines. For example, a triple Brix-value-QTL pyramided isogenic introgression line showed a 145% higher Brix yield compared to the original M82 tomato line, providing valuable material for breeding (Gur and Zamir, 2015; Prinzenberg et al., 2021; Wang Z et al., 2022).

Aroma is also an essential component of flavor quality. In a previous study, QTL mapping indicated that *SlFLORAL4* was the candidate gene for the phenylalanine-derived volatile locus on chromosome 4 in tomato, and the contents of 2-phenylethanol, phenylacetaldehyde, and volatile 1-nitro-2-phenylethane were significantly reduced in the CRISPR mutant of *SlFLORAL4* (Tikunov et al., 2020). This finding highlights the importance of utilizing natural variations for high aroma quality. Due to the relatively rich genetic basis of aroma, many elite lines with aroma have been developed (Tzin et al., 2015); thus, reverse genetic strategies, such as CRISPR, may offer a faster approach than forward genetic strategies (e.g., QTL mapping) for the production of high-quality breeding materials.

Regarding ornamental value, color is an important component of the traits which contributes to the ornamental value of terrace mini-horticultural landscapes. Since some painting pigments are extracted from natural plants, the potential exists to match colors artificially in living plants. The synthesis of some plant pigments has been genetically dissected and can be used in breeding procedures. As reported for tomatoes, CRISPR interruption of *SlPSY1*, *SlMYB12*, and *SlSGR1* interrupts the synthesis of carotenoids, naringenin chalcone, and chlorophyll, respectively, resulting in fruit color changes from red to yellow, pink, and brown (Yang et al., 2022). Triple mutants, with mutations in all three genes, exhibit a light green color; while double mutants, depending on the genes mutated, display light yellow, pink-brown, and yellow-green colors (Yang et al., 2022), thus demonstrating the ability to match new colors using basic pigments and genes (allelic variations) extracted from plant organs (Ye et al., 2022).

## 4 Pre-breeding for other types of terrace vegetables

### 4.1 Liana and fruit vegetables

For most liana and fruit vegetables, relatively taller plant architecture is the limitation factor which need to be improved for terrace cultivation.

For example, the *ER* orthologs *CmER*, *CsER*, and *CmoER* regulate internode length in melon, cucumber, and pumpkin, respectively (Torii et al., 1996; Xin et al., 2022). As predicted, the internode lengths of CRISPR-mutated genotypes *Cmer*, *Cser*, and *Cmoer* were 40%, 34%, and 60% shorter, respectively, compared to their parallel wild genotypes (Xin et al., 2022). The rare natural variation in the 5’ UTR of *CmoYABBY1*, known as the genetic essence of the *Bu locus*, results in a bushy architecture with clustered leaves and highly compressed internodes in the CRISPR mutant genotype *Cmoyabby1/bu*. This mutant genotype exhibits similar yield per plant to *CmoYABBY1* but significantly higher yield per square meter under high plant density conditions (Wang S et al., 2022). The suppression of stem length by *CmoYABBY1* variation is dose-dependent (Wang S et al., 2022), suggesting the potential value of *Cmoyabby1/bu* in adapting to different types of terraces structures. In the case of *CsTFL1* in cucumber, a non-synonymous SNP disrupts the interaction between *CsTFL1* and *CsNOT2a*, resulting in a loss of its ability to delay flowering (Wen et al., 2019). *CsTFL1* is expressed in the subapical regions of the shoot apical meristem, lateral meristem, and young stems, indicating its multiple effects on plant architecture. Knockdown of *CsTFL1* through RNAi leads to determinate growth and the formation of terminal flowers, resulting in a significant reduction in plant size, while flowering time remains unaffected (Wen et al., 2019). Although *CsTFL1* does not possess a CRISPR mutation, the natural non-synonymous allele can be utilized for breeding dwarf cucumbers using marker-assisted selection (MAS). Recent advancements in *Agrobacterium*-mediated transgenic and CRISPR technologies have enabled the genetic modification of an increasing number of *Cucurbitaceae* crops to achieve a compact plant size.

Kiwifruit, a newly domesticated climbing woody perennial liana crop, utilizes *CEN*-like genes as flowering time repressors (Varkonyi-Gasic et al., 2013; Voogd et al., 2017). CRISPR-induced bi-allelic mutations of *AcCEN4* and *AcCEN* in kiwifruit result in a compact annual plant with axillary inflorescences and rapid terminal flower and fruit development, making it suitable for terrace vegetable cultivation (Varkonyi-Gasic et al., 2018). Furthermore, a QTL on chromosome 26 increases vitamin C content in kiwifruit (McCallum et al., 2019), which help kiwifruit to a high nutrient food source for citizens.

For groundcherry, a related fruit vegetable crop of tomato, *PgER* is a member of the *ER* gene family that regulates stem length (Zhang et al., 2018; Kwon et al., 2019). The phenotype of the mutant *Pger* is more severely condensed compared to that of the tomato *Sler* mutant and resembles the *Slsp/Sp5g/Sler* triple mutant, while maintaining a similar fruit number and Brix content (Kwon et al., 2019). While, as for fruit crops like capsicum, the so-called chilli, the plant architecture is very suitable for terrace environment, and its flavor quality and nutrient quality has also been fit the scope of consumption of citizens, they could be adopted directly, the genetic improvement of capsicum in other traits is a kind of “add flowers to the brocade” for terrace cultivation.

### 4.2 Leafy vegetables

Leafy vegetables are very suitable for terrace cultivation for their dwarf or cramped architecture which fit the volume of terrace and cultivating facilities accordantly, including but not limited to lettuce, kale, broccoli, and bolt used rapeseed. The ornamental value and nutrient quality will become bonuses for these species if they could.

*Rll1* is a gene that induce the synthesis of anthocyanins, resulting in the red color of lettuce leaves (Su et al., 2019). Chlorophyll can also influence external traits, as observed in lettuce, where *LsVAR2* induces the formation of green speckles in the albino cotyledon (Nguyen et al., 2021). In kale, the CRISPR knockdown of *BoaCRTISO* results in the simultaneous reduction of chlorophyll and carotenoid concentrations. As a result, the leaf color changes from green to yellow, which weakens the color-masking effect of chlorophyll (Sun et al., 2020). In broccoli, a QTL mapping study indicates that two QTLs named *Pur7.1* and *Pur9.1* facilitate the biosynthesis of anthocyanin, and induce the purple cauliflower phenotype (Liu et al., 2022). Further, the CRISPR mutation of *BolMYB28* increases glucoraphanin content in the leaves (Kim et al., 2022), which induces a healthcare usage of broccoli. For rapeseed, the edible value is reflected through bolting, and the ornamental value is reflected through flowering. *PAP2* induces the synthesis of anthocyanins, resulting in the pink color of rapeseed petals, and *CCD4* induces carotenoid synthesis, resulting in yellow coloration in rapeseed petals (Ye et al., 2022). The combination of anthocyanins and carotenoids forms a new color, i.e., apricot flower petal in rapeseed via the co-expression of *CCD4* and *PAP2* (Ye et al., 2022). By the way, early bolting could be accomplished by MAS (Fang et al., 2022), which makes the citizens could harvest the bolts earlier.

Compared to other types of vegetable crops, the current deficiency is the lack of reported genetic research of flavor quality for leafy vegetables, and most researchers use cultivation skills, fertilizers, and equipment to enhance flavor quality (Silva and Zorzeto, 2019; Gangathilaka et al., 2022). However, the anticipated carbon peak in 2050 (Zheng et al., 2022) means that enhanced genetic strategies may be a more efficient means of saving energy and resources, thereby improving megacity sustainability. We believe that the development of pan-genomes will allow the discovery of more alleles for pre-breeding using CRISPR or MAS for terrace vegetables (Fei, 2019; Gao et al., 2019).

### 4.3 Root vegetables

Root vegetables are also very suitable for terrace cultivation, including but not limited to carrots, onions, or turnip. Taking carrots as an example, the plant height and width of carrot is perfect for terrace environment, and its nutrient quality is also excellent, and its ornamentally proper for terraces. In spite of this, carrots could be genetically improved for higher nutrient content and ornamental value. For instance, *DcMYB7* induces the synthesis of anthocyanin, resulting in the purple color of taproot (Xu et al., 2019). While the heterologous expression of *CYP76AD1*, *DODA1*, and *DOPA5GT* induces betalain in carrot, resulting in a red-violet color in the taproot (Deng et al., 2023). These genes not only increase the ornamental value of carrots, but also enhance medicinal and edible homologous functions. Generally, root vegetables expand the range of options available for terrace cultivation. Although some marker-free alleles of the mentioned genes above have yet to be identified, they hold the potential to serve as valuable pre-breeding resources.

### 4.4 Cereal crops

Flexibly consider, some cereal crops also suitable for terrace cultivation for their special identities of flavor or nutrient.

For instance, fresh maize can be eaten as vegetable in diets. In maize, the flavor compound 2-acetyl-1-pyrroline (2AP) is regulated by the activity of betaine aldehyde dehydrogenase 2 (BADH2). Natural maize varieties do not produce 2AP, but CRISPR-generated double mutants of *ZmBADH2a* and *ZmBADH2b* can produce 2AP in fresh and dry maize seeds, enhancing the aroma profile (Wang Y. et al., 2021).

Natural disasters, sudden epidemics, or uncertainties in human society can disrupt vegetable supply, leading to potential nutrient deficiencies. In such situations, nutrient enhancement or “biofortification” becomes crucial to ensure an adequate supply of important nutrients such as chlorophyll, cellulose, and vitamins from plants (Vlčko and Ohnoutková, 2019; Bhambhani et al., 2021), which is an important part of the resilience of megacities (Junior, 2017; Langemeyer et al., 2021). Just as the CRISPR mutation of *OsHOL1* increases iodine content (Carlessi et al., 2021), the CRISPR-mediated marker-free double insertion of *SSU-crtI* and *ZmPsy* has been shown to increase carotene content in rice (Dong et al., 2020). Further, as the CRISPR mutation of *TaIPK1* improves iron and zinc accumulation in wheat (Ibrahim et al., 2021), and the CRISPR mutation of *FtMYB45* promotes flavonoid biosynthesis in buckwheat (Wen et al., 2022).

## 5 Hybrid vegetable seeds are encouraged for terrace cultivation

Although the basic three prerequisites are the basic requirements of the cultivars, sufficient yield is also need to be noted, especially for the smaller architecture plants. Sometime, it is not realistic to expect smaller plants modified for cramped conditions to produce comparable yields to those of stronger, taller plants grown in larger spaces. To address this issue, on one hand, the existed heterotic patterns should be insisted; on the other hand, we propose a single-gene advantageous stacking strategy to increase fruit yield in cramped plants (Fang et al., 2022). For instance, several gene families, including *IAA7*, *FLC*, *TFL*, *SFT*, and *SSP*, have been shown to have significant effects on plant architecture and exhibit strong single-gene heterosis, leading to increased yield (Krieger et al., 2010; Guo et al., 2014; Park et al., 2014; Li et al., 2019; Fang et al., 2022). Heterozygous genotypes of these genes can be stacked using polycistronic CRISPR or QTL pyramiding in hybrid breeding systems (Fang et al., 2022). Conversely, some high-yield-related alleles are effective in the homozygous state and can be stacked in elite lines to compensate for the reduced yields of smaller plants (Gur and Zamir, 2015; Song et al., 2021; Wang S et al., 2022).

## 6 Summary and expectation

**Figure 1:**
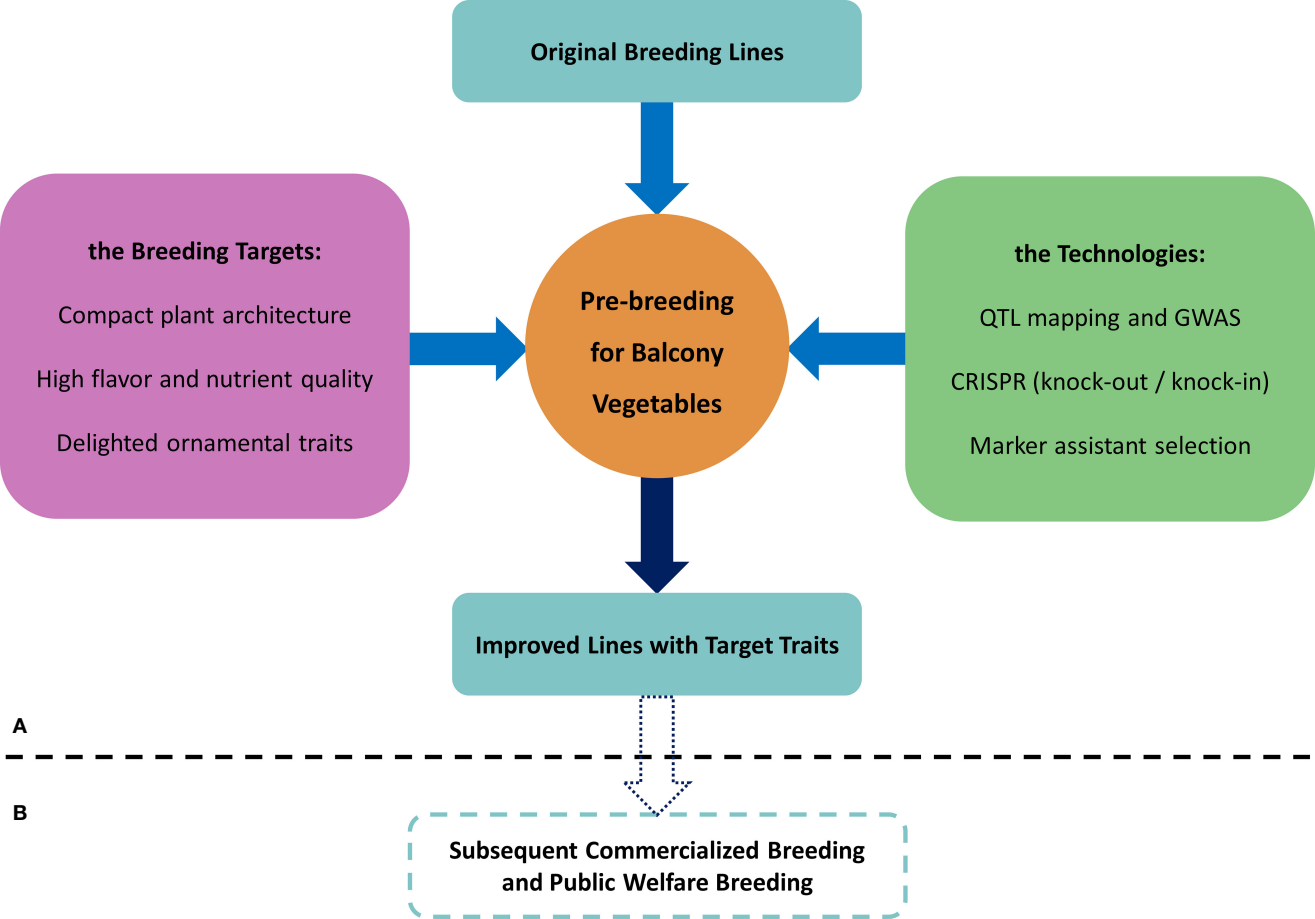
The thumbnail of pre-breeding. **(A)** Mind mapping of pre-breeding for terrace vegetables. **(B)** Subsequent steps after pre-breeding.

In conclusion, the terrace vegetable system is a broad concept, and traditional food and oil crops, vegetables, and fruits are likely to become specialized commercial varieties for terrace vegetable systems after genetic improvement, provided that the plant materials meet the three main requirements described in this review (**Figure 1**; **Supplementary Table 2**) i.e., the breeders and citizens could choose their own vegetable crops following the information including but not limited to **Supplementary Table 1**. Furthermore, we recognize that the terrace vegetable system can provide several benefits. It offers flexibility in the supply of fresh vegetables, ensuring the availability of high-quality and nutritious food for urban populations. Additionally, it contributes to improving the microecological environment of urban residents, enhancing their well-being. Moreover, it increases the ornamental value of fruits and vegetables, adding to the aesthetic appeal of urban landscapes. Finally, it provides opportunities for citizen recreation, which can be considered a luxury in megacities.

A major constraint is that parts of the citizens tend to get access to the seeds free of cost, even when the seeds are unknown pedigrees or segregating individuals of hybrids. To solve this problem which might hinder the development of the seed industry for terrace cultivation, on one hand, the breeders could enhance the level of breeding procedures and produce high performance seeds to attract citizens to buy; on the other hand, breeders could host some public activities to donate some seeds to the citizens as welfares. Our pre-breeding work strategy is a pivotal step to produce elite parental lines not only for commercialized breeding but also for welfare breeding.

In spring 2021, the vegetable garden owned by the first author of this review received attention from the public and media at the 10th China Flower EXPO and won a prize for scientific and technological innovation. This highlights that the selection and breeding of vegetable varieties suitable for special urban planting environments meet the current demand for urban green sustainable development and the needs of people living in cities. Thus, pre-breeding of terrace vegetables have forward-thinking and commercial application value. Only a few vegetable types have been genetically dissected, and no commercial varieties have been specifically bred for terrace vegetables. However, pre-breeding programs provide several breeding lines for subsequent steps, showing potential for yielding the most suitable vegetable varieties in the near future and informing important future research on gene function. With the development of 5G breeding (Varshney et al., 2020), more horticultural crops and even field crops could be genetically improved to fulfill the scope of terrace cultivation of vegetable crops. Thus, genetic improvement and the use of pre-breeding will not only support a promising industry but also enhance the nutrient resilience of megacities (**Supplementary Figure 1**).

## Author contributions

KM and CF wrote the manuscript. YY helped to revise the manuscript. CF and KM designed the study. KM shared the experience. CF prepared the figures. All authors contributed to the article and approved the submitted version.
