# Supplementary material for: Mainstreaming production and nutrient resilience of vegetable crops in megacities: pre-breeding for terrace cultivation
Source: Front Plant Sci. 2023 Nov 17;14:1237099. doi: 10.3389/fpls.2023.1237099 (PMC10694833; doi:10.3389/fpls.2023.1237099)
Supplement: Supplementary file 1 [file Presentation_1.pdf]

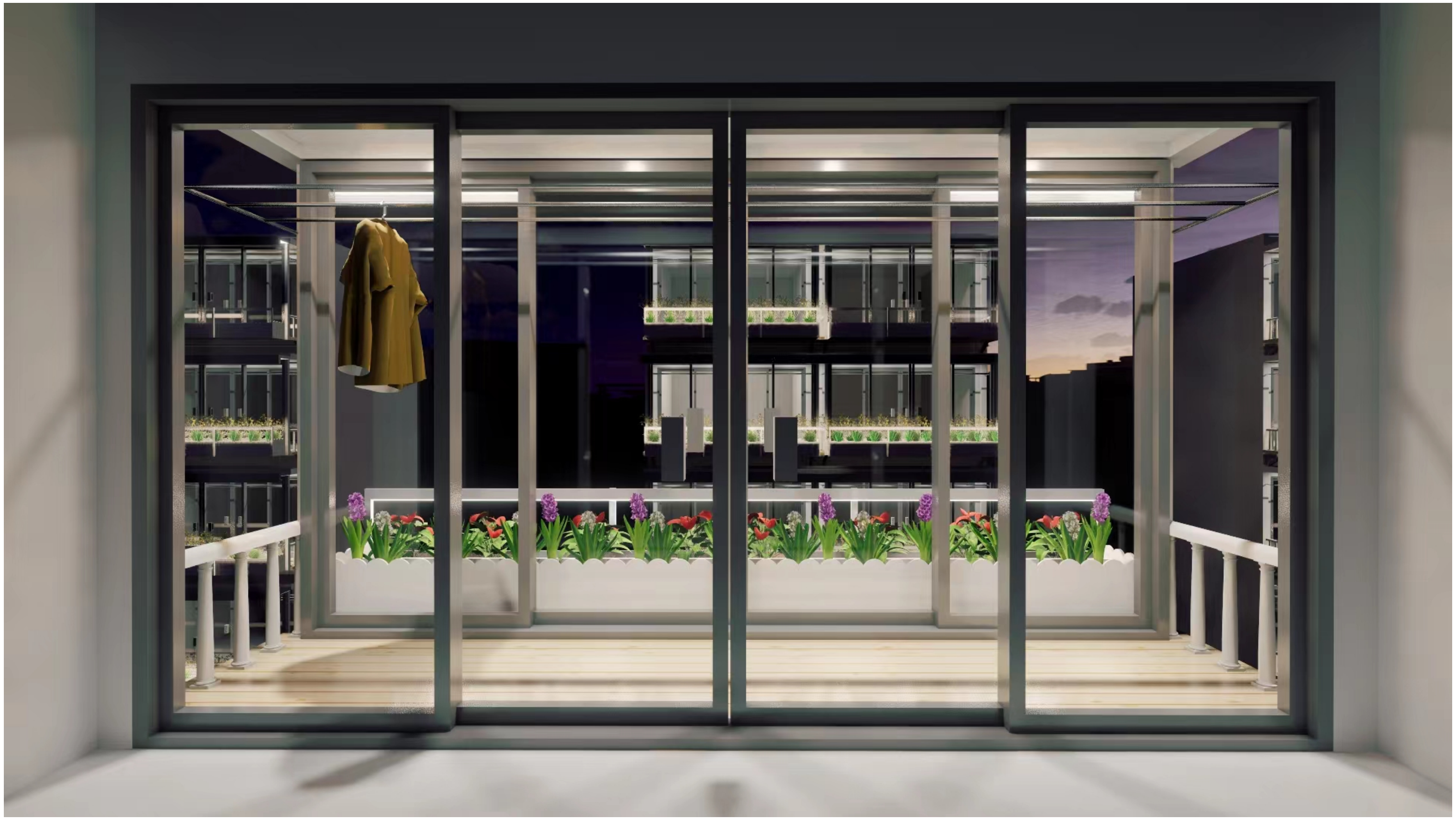

Supplementary Figure 1 | A case of terrace cultivation to show a promising industry.

The plants used in this figure were just for art effects, the citizens need to choose suitable plants following Supplementary Table 1 and 2 of this review according to their own terraces, balconies or roofs.

Supplementary Table 1 Advantages and dificiency of different crops for terrace cultivation

crop species	plant architecture	nutrient or flavor quality	oranmental value
tomato	○	○	○
groundcherry	○	○	○
cucumber	×	○	○
pumpkin	×	○	○
kiwifruit	×	√	○
watermelon	○	√	○
melon	×	○	○
capsicum	√	√	√
broccoli	√	○	○
kale	√	○	○
cabbage	√	○	○
lettuce	√	○	○
rapeseed	○	○	○
carrot	√	○	○
onion	√	√	○
turnip	√	√	○
fresh maize	√	○	○
rice	○	×	○
wheat	○	×	○
buckwheat	○	×	○
garlic	√	√	○
taro	○	○	○

Note: √ means very suitable natually, ○ means alternative, × means not suitable enough and need to be improved.

Supplementary Table 2 Genes for pre-breeding for terrace cultivation

crop species	gene or QTL names	technology for target alleles	simplified description of gene functions	reference
broccoli	*BolMYB28*	CRISPR	glucoraphanin synthesis	Kim Y et al 2022
buckwheat	*FtMYB45*	CRISPR	flavonoid synthesis	Wen et al 2022
carrot	*DcMYB7*	*	anthocyanin synthesis	Xu et al 2019
carrot	*CYP76AD1*	*	betalain synthesis	Deng et al 2023
carrot	*DODA1*	*	betalain synthesis	Deng et al 2023
carrot	*DOPA5GT*	*	betalain synthesis	Deng et al 2023
cucumber	*CsER*	CRISPR	internode length	Xin et al 2022
cucumber	*CsTFL1*	QTL	flowering	Wen et al 2019
groundcherry	*PgER*	CRISPR	internode length	Kwon et al 2019
kale	*BoaCRTISO*	CRISPR	ehlorophy and carotenoid synthesis	Sun et al 2020
kiwifruit	*AcCEN4*	CRISPR	internod length and flowering	Varkonyi et al 2013; Voogd 2017; Varkonyi et al 2018
kiwifruit	*AcCEN*	CRISPR	internod length and flowering	Varkonyi et al 2013; Voogd 2017; Varkonyi et al 2018
kiwifruit	*qVITAMIN.chr2*	QTL	vitamin C	McCallum et al 2019
lettuce	*Rll1*	*	anthocyanin synthesis	Su et al 2019
lettuce	*LsVAR2*	*	Chlorophyll synthesis	Nguyen et al 2021
maize (fresh)	*ZmBADH2a*	CRISPR	2-acetyl-1-pyrroline synthesis	Wang Y et al 2021
maize (fresh)	*ZmBADH2b*	CRISPR	2-acetyl-1-pyrroline synthesis	Wang Y et al 2021
melon	*CmER*	CRISPR	internode length	Xin et al 2022
pumpkin	*CmoER*	CRISPR	internode length	Torii et al 1996
pumpkin	*CmoYABBY1 / Bu locus*	QTL, CRISPR	bushy and internode length	Wang S et al 2022
rapeseed	*PAP2*	CRISPR	anthocyanin synthesis	Ye et al 2022
rapeseed	*CCD4*	CRISPR	carotenoid synthesis	Ye et al 2022
rice	*OsHOL1*	CRISPR	iodine content	Carlessi et al 2021
rice	*SSU-crtI*	CRISPR knock-	carotene content	Dong et al 2020
rice	*ZmPsy*	CRISPR knock-	carotene content	Dong et al 2020
tomato	*SlSP*	QTL, CRISPR	flowering and indeterminate growth	Kwon et al 2019
tomato	*SlSP5G*	CRISPR	flowering	Kwon et al 2019
tomato	*SLER*	CRISPR	internode length	Kwon et al 2019
tomato	*SlINVINH1*	CRISPR	glucose, fructose, and brix	Wang B et al 2021; Kavaguchi et al 2021
tomato	*SlVPE5*	CRISPR	glucose, fructose, and brix	Wang B et al 2021; Kavaguchi et al 2021
tomato	*qBRIX.chr9*	GWAS	brix content	Kim et al 2021
tomato	*qBRIX.chr2*	QTL	brix content	Prinzenberg et al 2021; Wang Z et al 2021; Gur and Zamir 2015
tomato	*qBRIX.chr6*	QTL	brix content	Prinzenberg et al 2021; Wang Z et al 2021; Gur and Zamir 2015
tomato	*SlFLORAL4*	QTL; CRISPR	phenylalanine synthesis	Tikunov et al 2020
tomato	*SlPSY1*	CRISPR	caroteniod synthesis	Yang et al 2022
tomato	*SlMYB12*	CRISPR	naringenin chalcone synthesis	Yang et al 2022
tomato	*SlSGR1*	CRISPR	chlorophyll synthesis	Yang et al 2022
wheat	*TaIPK1*	CRISPR	zinc accumulation	Ibrahim et al 2021

Note: The excellent alleles of target genes could be introduced into breeding lines by marker assisted selection (MAS). "*" indicates the ideal alleles need to be identified or manipulated.
